# FN-EDA mediates angiogenesis of hepatic fibrosis via integrin-VEGFR2 in a CD63 synergetic manner

**DOI:** 10.1038/s41420-020-00378-9

**Published:** 2020-12-08

**Authors:** Xiaonan Su, Xiaowen Ma, Xiaoyu Xie, Hao Wu, Le Wang, Yuemin Feng, Zhen Yu, Chenxi Liu, Jianni Qi, Qiang Zhu

**Affiliations:** 1 Department of Gastroenterology, Shandong Provincial Hospital, Cheeloo College of Medicine, Shandong University, Jinan, Shandong China; 2 Shandong Provincial Engineering and Technological Research Center for Liver Diseases Prevention and Control, Jinan, 250021 Shandong China; 3 Department of Gastroenterology, Shandong Provincial Hospital Affiliated to Shandong First Medical University, Jinan, Shandong China; 4 Department of Central Laboratory, Shandong Provincial Hospital Affiliated to Shandong First Medical University, Jinan, 250021 Shandong China

**Keywords:** Liver fibrosis, Liver cirrhosis

## Abstract

Pathological angiogenesis is an important component of hepatic fibrosis along with fibrous deposition, but its role is not well understood. Here, we demonstrated that fibronectin containing extra domain A(FN-EDA), a fibronectin splice variant highly expressed in hepatic fibrosis, mediated angiogenesis in disease progression. FN-EDA was positively correlated with pathological angiogenesis in hepatic fibrosis, and a reduction in FN-EDA expression was associated with diminished intrahepatic angiogenesis and fibrosis. FN-EDA mostly colocalized with hepatic stellate cells (HSCs) and interference or blockage of FN-EDA attenuated migration and tube formation in co-cultured endothelial cells. Mechanistic studies indicated that FN-EDA was secreted to promote phosphorylation of VEGFR2 with the assistance of integrin and CD63. Targeting FN-EDA-integrin combination postponed the progression of hepatic angiogenesis and fibrosis in vivo. These results indicated that FN-EDA plays an emerging role in angiogenesis in hepatic fibrosis and could be a potential therapeutic intervention for the disease.

## Introduction

Hepatic fibrosis is a successive process that is accompanied by excessive deposition of extracellular matrixes (ECM) and pathological angiogenesis, which is frequently observed in patients with chronic liver diseases^1^. Without proper and timely intervention, hepatic fibrosis gradually tends to become hepatic cirrhosis, one of the most common lethal diseases worldwide^2^. Recently, increasing evidence have indicated that intrahepatic pathological angiogenesis with an aberrant angioarchitecture is an indispensable part of hepatic fibrogenesis^3,4^. Pathological angiogenesis triggered by vascular endothelial growth factor (VEGF) overproduction is believed to be central to liver fibrosis progress and the development of portal hypertension^5–7^. VEGF/VEGFR2 signaling is essential in angiogenesis and the crosstalk between hepatocytes and hepatic sinusoidal endothelial cells (HSECs), some studies even suggest VEGFR2 inhibitor Bevacizumab could attenuate hepatic fibrosis^8–12^.

Fibronectin (FN) is a high-molecular-weight multifunctional glycoprotein whose pre-mRNA has three alternative splicing sites (extra domain A (EDA), extra domain B (EDB), and type III homology connecting segment (IIICS)) which generates twenty different isoforms of the FN protomer. Traditionally, circulating soluble plasma FN (pFN) lacks both the EDA and EDB segments secreted by hepatocytes, while cellular FN (cFN) contains variable proportions of EDA or EDB or both which are enriched in the extracellular matrix^13–15^. FN-EDA and FN-EDB are expressed nearly ubiquitously in embryonic tissues and are associated with cardiovascular development^16–18^ while their expressions are strictly limited in normal adult tissues but is increased in various pathological states. Accumulated evidence has demonstrated that FN-EDA participates in some fibrotic diseases of many organs, including the dermis, lung and bone marrow^19–21^, and several physiopathologic processes such as intimal proliferation, wound healing and ischemia reperfusion injury^22–25^.

The explicit role of FN-EDA in hepatic fibrosis is still controversial. Previous studies reported that FN-EDA is upregulated by TGF-β in hepatic fibrosis model^26^, and male FN-EDA KO mice are protected from CCl_4-_induced hepatic fibrosis^27^. However, an in vivo study indicated that total fibronectin is dispensable for hepatic fibrogenesis^28^. In addition, although some reports suggest that FN-EDA promotes HSC activation^29^, others found that FN-EDA could promote only HSC motility but not differentiation^27^. FN-EDA participates in hepatic fibrosis but has a limited effect on fibrogenesis. Our previous works have demonstrated that the expression of FN is elevated in HSCs via the LPS/TLR4 pathway in a mouse liver fibrosis model, and we preliminary verified its association with vascular changes^30^. Therefore, considering our previous works and that FN-EDA participates in embryonic vascular morphogenesis and retinal neovascularization^18,31^, we propose that FN-EDA may mediate pathological angiogenesis in hepatic fibrosis.

Herein, we investigated the expression of FN-EDA and analyzed its relationship with intrahepatic angiogenesis in hepatic fibrosis patients and a CCl_4_-induced mouse hepatic fibrosis model. Mechanistically, we demonstrated that FN-EDA secreted from HSCs promoted pathological angiogenesis by activating the VEGFR2 pathway in endothelial cells with the assistance of integrin and CD63 in a paracrine manner. Meanwhile, we preliminarily evaluated the potential therapeutic effect of blocking FN-EDA in vivo. Our studies identified a new molecular mechanism of FN-EDA on pathological angiogenesis and provided a potential target for therapeutic interventions in hepatic fibrosis.

## Results

### FN-EDA expression was elevated in hepatic fibrosis and positively correlated with angiogenesis

**Fig. 1:**
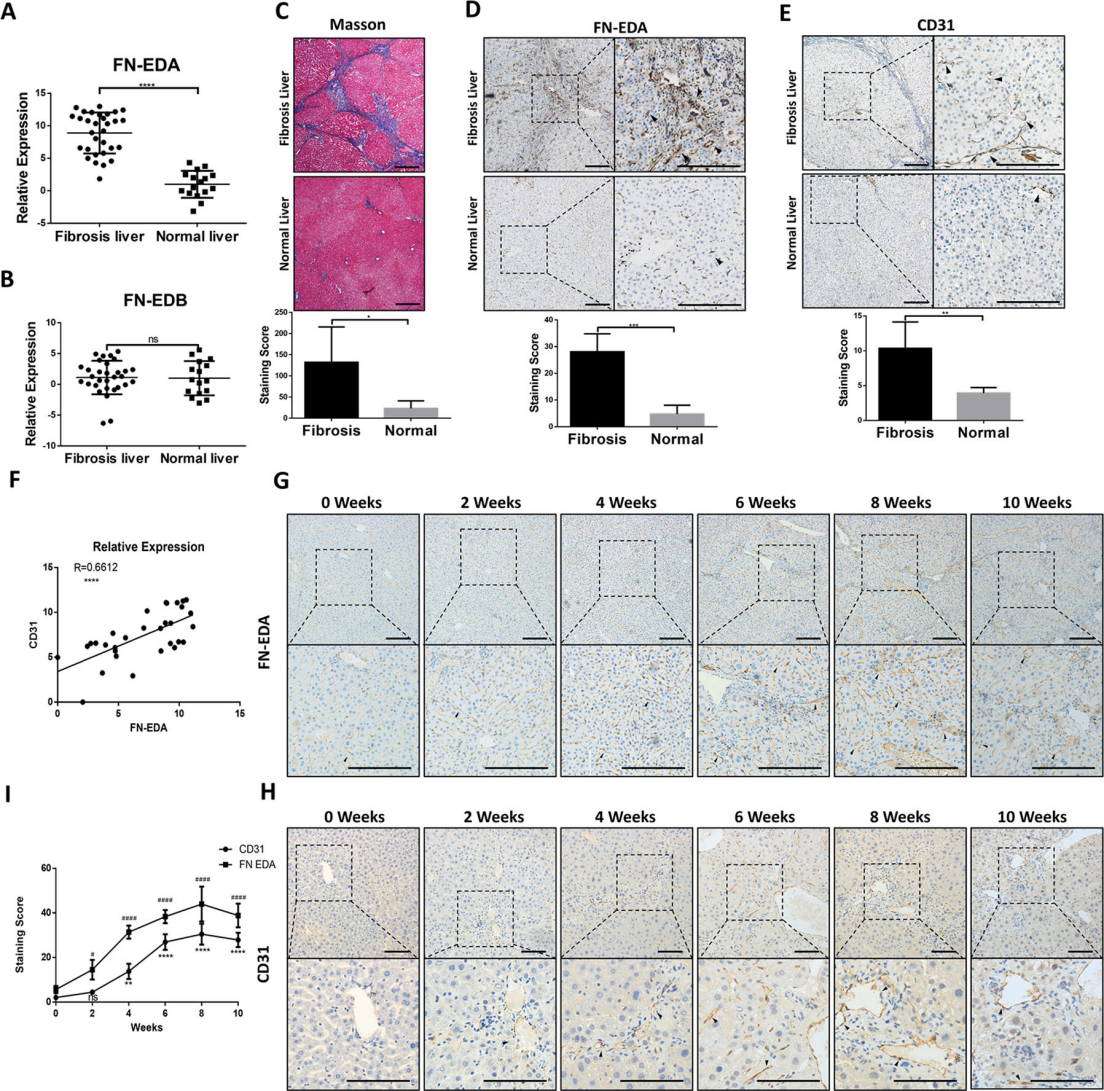
Expression of FN-EDA is associated with CD31 in hepatic fibrosis. **A**, **B** qRT-PCR analysis of FN-EDA and FN-EDB mRNA levels between liver tissue of CHD patients (*n* = 30) and healthy control (*n* = 15). (GAPDH was used as an internal control. Data are shown as the mean ± SEM. FN-EDA: ****P < 0.0001; FN-EDB: P: ns) **C** Masson staining in human fibrosis hepatic tissues (*n* = 5) and normal tissues (*n* = 5). (quantification relative area of Masson staining. Data are shown as mean ± SEM. *P = 0.0217. Scale bar = 200 μm. **D** Immunohistochemical staining of FN-EDA in human fibrosis hepatic tissues (*n* = 5) and normal tissues (*n* = 5). (Data are shown as mean ± SEM. ***P = 0.0001. Scale bar = 200 μm). **E** Immunohistochemical staining of CD31 in human fibrosis hepatic tissues (*n* = 5) and normal tissues (*n* = 5). (Data are shown as mean ± SEM. **P = 0.0060. Scale bar = 200 μm). **F** qRT-PCR analysis of correlation between FN-EDA and CD31 in human fibrosis hepatic tissues (*n* = 30). (*R* = 0.6612 ****P < 0.0001) **G**, **H** FN-EDA and CD31 staining in livers of CCl4-treated mice biweekly until tenth weeks (*n* = 18) (Scale bar = 200 μm; arrows: staining) **I** Analysis of FN-EDA and CD31 expression in IHC (*n* = 3).

Our previous study demonstrated that FN is overexpressed in HSCs in mouse hepatic fibrosis. As EDA and EDB are the segments of FN unique to pathological states and are independently expressed due to alternative splicing^32,33^, we first detected their expression in normal and fibrotic livers. qRT-PCR analysis showed that EDA expression was significantly increased in the fibrotic group versus the healthy group (Fig. 1A) while the expression of EDB was not significantly different between the two groups (Fig. 1B). Thus, we obtained human hepatic sections and detected the expression of FN-EDA. Masson staining showed the differences in collagen deposition between patients and healthy individuals (Fig. 1C), and immunohistochemical analysis suggested that FN-EDA expression was significantly stronger in human fibrotic hepatic tissues than in normal tissues (Fig. 1D). We observed FN-EDA expression mainly in the Disse space, indicating that FN-EDA may be mechanistically involved in the biological functions of endothelial cells.

Therefore, we further explored the relationship between FN-EDA and pathological angiogenesis in liver fibrosis. First, we detected CD31 (a reliable marker for angiogenesis) expression in the above hepatic tissues and found that CD31 was highly expressed in fibrotic livers (Fig. 1E). Meanwhile, FN-EDA expression was positively correlated with CD31 in human fibrosis liver samples by qRT-PCR analysis (Fig. 1F). Furthermore, we produced CCl_4_-treated mice, harvested hepatic tissue and evaluated the expression of FN-EDA and CD31 in these tissues at the histological level biweekly until ten weeks. We found that both FN-EDA and CD31 expression were elevated over time with CCl_4_ treatment especially in the first 8 weeks and were significantly higher than at the beginning (Fig. 1G–I). The above results suggested that FN-EDA was positively correlated with angiogenesis in hepatic fibrosis.

### FN-EDA derived from HSCs promoted pathological angiogenesis in hepatic fibrosis

**Fig. 2:**
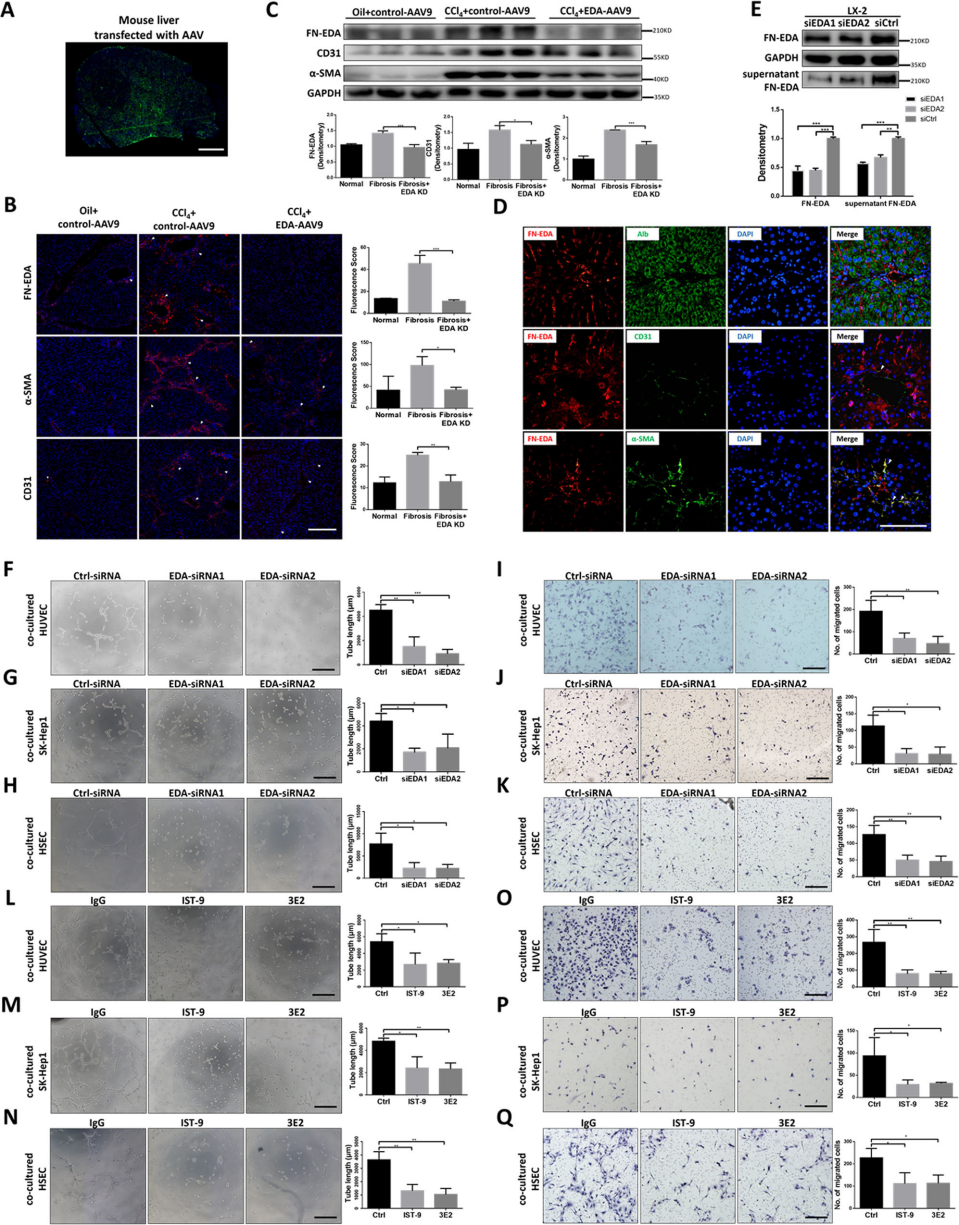
FN-EDA promoted angiogenesis in vivo and vitro. **A** Overall view of mouse liver infected with AAV9 (Green: EGFP). Six-week-old male wild-type C57BL/6 mice were intraperitoneally injected with 20% CCl_4_ dissolved in olive oil, or olive oil alone twice a week (2 ml/kg) for 8 weeks. AAV9 control vector (AAV9-Ctrl) or AAV9-FN-EDA-shRNA were injected into mice through tail vein at the beginning of week 1 and week 5. Mouse livers were harvest at the end of week 8. (Scale bar = 2.5 mm.) **B** Immunofluorescence staining of FN-EDA, α-SMA and CD31 in AAV9-FN-EDA-shRNA or AAV9-Ctrl infected mice with experimental hepatic fibrosis (*n* = 3). (Arrows: staining. Data are shown as mean ± SEM. FN-EDA: ***P < 0.0001; α-SMA: *P < 0.01; CD31: **P < 0.001. Scale bar = 200 μm.) **C** Immunoblot analysis of FN-EDA, α-SMA and CD31 in AAV9-FN-EDA-shRNA or AAV9-Ctrl infected mice with experimental hepatic fibrosis (n = 3). (Data are shown as mean ± SEM. α-SMA: ***P < 0.0001; FN-EDA: ***P < 0.0001; CD31: **P < 0.001.) **D** Mouse experimental hepatic fibrosis samples were processed for immunofluorescence co-staining for FN-EDA (red) with α-SMA (activated HSC marker), CD31 (endothelial marker), and Alb (hepatocyte marker) (green). Nucleus were counterstained with DAPI. (Arrows: co-staining. Scale bar = 100 μm.) **E** Immunoblot result of FN-EDA in LX-2 cells and its supernatant. LX-2 cells were transfected with two FN-EDA-siRNAs or Ctrl-siRNA following the general procedure. Serum-free DMEM was replaced for 24 h culturing and then collected supernatant and add loading buffer for immunoblot. (Data are shown as mean ± SEM; FN-EDA: EDA-siRNA1 versus Ctrl, ***P < 0.0001; EDA-siRNA2 versus Ctrl, ***P < 0.0001; supernatant FN-EDA: EDA-siRNA1 versus Ctrl, ***P < 0.0001; EDA-siRNA2 versus Ctrl, **P < 0.001.) **F**–**H** Tube formation assay of HUVECs, SK-hep1 cells and HSECs co-cultured with LX-2 cells for 6 h. LX-2 cells were transfected with two FN-EDA-siRNAs or Ctrl-siRNA (*n* = 3). (Data are shown as mean ± SEM; HUVEC: EDA-siRNA1 versus Ctrl, **P < 0.001; EDA-siRNA2 versus Ctrl, ***P < 0.0001; SK-hep1: EDA-siRNA1 versus Ctrl, *P < 0.01; EDA-siRNA2 versus Ctrl, *P < 0.01. HSEC: EDA-siRNA1 versus Ctrl, *P < 0.01; EDA-siRNA2 versus Ctrl, *P < 0.01. Scale bar = 100 μm.) **I**–**K** Migration assay of HUVECs, SK-hep1 cells and HSECs co-cultured with LX-2 for 24 h (*n* = 3). (Data are shown as mean ± SEM; HUVECs: EDA-siRNA1 versus Ctrl, *P < 0.01; EDA-siRNA2 versus Ctrl, ***P < 0.0001; SK-hep1 cells: EDA-siRNA1 versus Ctrl, *P < 0.01; EDA-siRNA2 versus Ctrl, *P < 0.01; HSECs: EDA-siRNA1 versus Ctrl, **P < 0.001; EDA-siRNA2 versus Ctrl, **P < 0.001; scale bar = 100 μm.) **L**–**N** Tube formation assay of HUVECs, SK-hep1 cells and HSECs co-cultured with LX-2 for 6 h. FN-EDA specific neutralizing antibodies IST-9 or 3E2 or mouse IgG (40 μg/ml) were pretreated for 0.5 h (*n* = 3). (Data are shown as mean ± SEM; HUVEC: IST-9 versus Ctrl, *P < 0.01; 3E2 versus Ctrl, *P < 0.01; SK-hep1: IST-9 versus Ctrl, *P < 0.01; 3E2 versus Ctrl, **P < 0.001. HSEC: IST-9 versus Ctrl, **P < 0.001; 3E2 versus Ctrl, **P < 0.001, scale bar = 100 μm.) **O**–**Q** Migration assay of HUVECs, SK-hep1 cells and HSECs co-cultured with LX-2 for 24 h. FN-EDA specific neutralizing antibodies IST-9 or 3E2 or mouse IgG (40 μg/ml) was pretreated for 0.5 h (*n* = 3). (Data are shown as mean ± SEM; HUVEC: IST-9 versus Ctrl, **P < 0.001; 3E2 versus Ctrl, **P < 0.001; SK-hep1: IST-9 versus Ctrl, *P < 0.01; 3E2 versus Ctrl, **P < 0.001; HSEC: IST-9 versus Ctrl, *P < 0.01; 3E2 versus Ctrl, *P < 0.01, scale bar = 100 μm).

To determine whether FN-EDA promoted angiogenesis in hepatic fibrosis, we produced FN-EDA knockdown mice through tail vein injection using a recombinant EDA-AAV9 vector and then treated the mice with CCl_4_ for 8 weeks. EGFP fluorescence showed the transfection efficiency in mouse livers (Fig. 2A), and the expression of FN-EDA was significantly decreased (Fig. 2B, C). FN-EDA KD mice had diminished neovessel density, CD31 expression and fibrosis level compared with the control group (Fig. 2B, C). The above results indicated that FN-EDA mediated pathologic angiogenesis in hepatic fibrosis to some extent. Next, we defined the cellular sources of FN-EDA during chronic hepatic fibrosis. We examined the colocalization of FN-EDA with albumin (hepatocyte marker), CD31 (endothelial marker), and α-SMA (activated HSC marker) in mouse fibrosis hepatic tissues. The expression of FN-EDA largely overlapped with that of α-SMA, slightly overlapped with that of CD31 and did not overlap with that of albumin (Fig. 2D), indicating that FN-EDA was mainly expressed in HSCs during chronic hepatic fibrosis.

To further determine how FN-EDA participates in pathological angiogenesis, we first detected the expression of FN-EDA in LX-2 cells in vitro. Consistent with expectations, FN-EDA was highly detected in LX-2 and its supernatant and was decreased after knockdown by two different FN-EDA-siRNAs (Fig. 2E). Then, we used a transwell plate to coculture LX-2 cells with several endothelial cells, including HUVECs (a standard endothelial cell line), SK-hep1 cells (a liver derived endothelial cell line) and primary HSECs. Tube formation and migration of endothelial cells were significantly decreased after knocking down FN-EDA in LX-2 cells (Fig. 2F–K). To further determine whether FN-EDA itself could activate endothelial cells in a direct manner, we used two different FN-EDA specific neutralizing antibodies, IST-9 and 3E2, to block FN-EDA in the above coculture environment. Tube formation and migration of endothelial cells promoted by LX-2 were also significantly attenuated (Fig. 2L–Q). In summary, all the results indicated that HSC derived FN-EDA promoted pathological angiogenesis in hepatic fibrosis in a paracrine manner.

### FN-EDA activated VEGFR2 phosphorylation not completely dependent on VEGFA

**Fig. 3:**
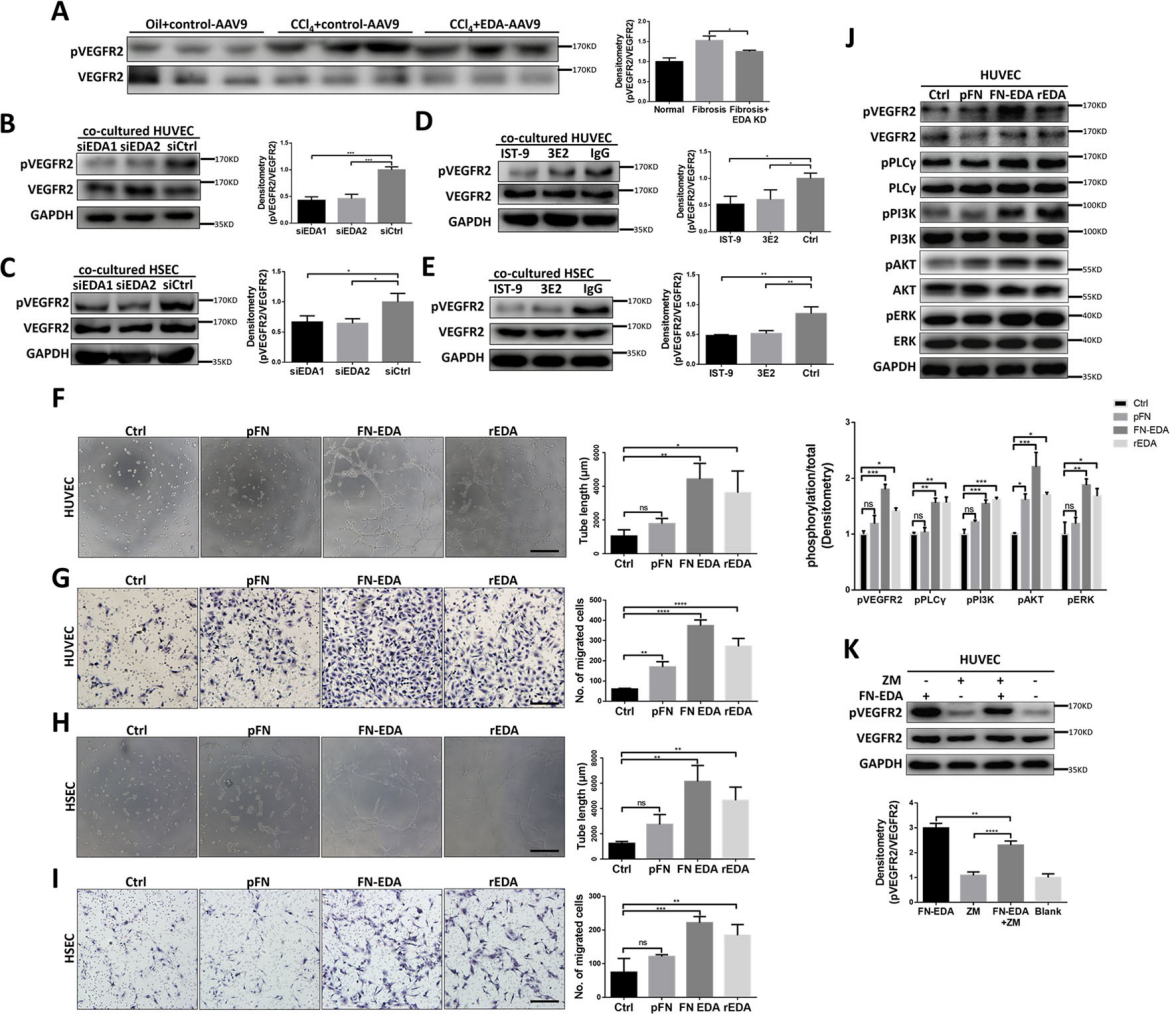
FN-EDA promoted phosphorylation of VEGFR2 in vivo and vitro. **A** Immunoblot result of pVEGFR2 and total VEGFR2 in AAV9-FN-EDA-shRNA or AAV9-Ctrl infected mice with experimental hepatic fibrosis (*n* = 3). (Data are shown as mean ± SEM; *P < 0.01.) **B**, **C** Immunoblot result of pVEGFR2 in HUVECs and HSECs co-cultured with FN-EDA-siRNAs or Ctrl-siRNA transfected LX-2 cells for 8 h (*n* = 3). (Data are shown as mean ± SEM; HUVEC: EDA-siRNA1 versus Ctrl, ***P < 0.0001; EDA-siRNA2 versus Ctrl, **P < 0.0001; HSEC: EDA-siRNA1 versus Ctrl, *P < 0.01; EDA-siRNA2 versus Ctrl, *P < 0.01.) **D**, **E** Immunoblot result of pVEGFR2 in HUVECs and HSECs co-cultured with LX-2 for 8 h. LX-2 were pretreated with neutralizing antibodies IST-9 or 3E2 or mouse IgG (40 μg/ml) for 0.5 h (*n* = 3). (Data are shown as mean ± SEM; HUVEC: IST-9 versus Ctrl, *P < 0.01; 3E2 versus Ctrl, *P < 0.01; HSEC: IST-9 versus Ctrl, **P < 0.001; 3E2 versus Ctrl, **P < 0.001.) **F** Tube formation assay of HUVECs treated with pFN, FN-EDA or rEDA (40 μg/ml) for 6 h (*n* = 3). (Data are shown as mean ± SEM; rEDA versus Ctrl: *P < 0.01; FN-EDA versus Ctrl: **P < 0.001; pFN versus Ctrl: ns. Scale bar = 100 μm.) **G** Migration assay of HUVECs treated with pFN, FN-EDA or rEDA (40 μg/ml) for 24 h. (Data are shown as mean ± SEM, *n* = 3. rEDA versus Ctrl: ****P < 0.00001; FN-EDA versus Ctrl: ****P < 0.00001; pFN versus Ctrl: **P < 0.001. Scale bar = 100 μm.) **H** Tube formation assay of HSECs treated with pFN, FN-EDA or rEDA (40 μg/ml) for 6 h (*n* = 3). (Data are shown as mean ± SEM; rEDA versus Ctrl: **P < 0.001; FN-EDA versus Ctrl: **P < 0.001; pFN versus Ctrl: ns. Scale bar = 100 μm.) I Migration assay of HSECs treated with pFN, FN-EDA or rEDA (40 μg/ml) for 24 h. FN-EDA and rEDA significantly promote HSECs tube formation (*n* = 3). (Data are shown as mean ± SEM; rEDA versus Ctrl: *P < 0.01; FN-EDA versus Ctrl: **P < 0.001; pFN versus Ctrl: ns. Scale bar =100 μm.) **J** Immunoblot result of VEGFR2 related pathway in HUVECs treated with pFN, FN-EDA or rEDA (40 μg/ml) for 6 h. (Data are shown as mean ± SEM; pVEGFR2: rEDA versus Ctrl: *P < 0.01; FN-EDA versus Ctrl: ***P < 0.0001; pFN versus Ctrl: ns; pPLCγ rEDA versus Ctrl: **P < 0.001; FN-EDA versus Ctrl: **P < 0.001; pFN versus Ctrl: ns; pPI3K rEDA versus Ctrl: ***P < 0.0001; FN-EDA versus Ctrl: ***P < 0.0001; pFN versus Ctrl: ns; pAKT rEDA versus Ctrl: *P < 0.01; FN-EDA versus Ctrl: ***P < 0.0001; pFN versus Ctrl: *P < 0.01; pERK rEDA versus Ctrl: *P < 0.01; FN-EDA versus Ctrl: **P < 0.001; pFN versus Ctrl: ns.) **K** Immunoblot result of pVEGFR2 in HUVECs. HUVECs were treated with FN-EDA (40 μg/ml) and/or ZM323881 (5 μM) for 6 h (*n* = 3). (Data are shown as mean ± SEM; FN-EDA versus FN-EDA + ZM323881, **P < 0.001; FN-EDA + ZM versus ZM323881, ****P < 0.00001).

Continuous hyperactivation of the VEGFR2 related pathway is considered the most critical aspect of pathological angiogenesis during chronic hepatic fibrosis^34^, so we examined the phosphorylation of VEGFR2 after knocking down and blocking FN-EDA. Decreasing phosphorylation levels of VEGFR2 were observed in FN-EDA KD mice comparing with fibrotic control (Fig. 3A), and knocking down of FN-EDA in LX-2 and blocking FN-EDA by neutralizing antibodies in vitro, decreased level phosphorylation levels of VEGFR2 was observed in co-cultured HUVEC and HSEC (Fig. 3B–E). To further determine whether the EDA segment itself plays a decisive role, we used recombinant EDA (rEDA), FN-EDA and pFN (EDA lacking FN) to treat HUVECs and HSECs, respectively. FN-EDA and rEDA but not pFN significantly enhanced the motility and tube formation of HUVECs and HSECs (Fig. 3F–I). Meanwhile, the phosphorylation of VEGFR2 and its downstream pathways including PI3K, AKT, PLCγ, and ERK were dramatically increased after stimulation by FN-EDA and rEDA compared with the control (Fig. 3J). A previous study suggested that FN-EDA increases VEGF-C expression in colorectal carcinoma^35^, so we considered whether this FN-EDA influenced VEGFR2 phosphorylation was VEGF dependent. We pretreated FN-EDA-stimulated HUVECs with the inhibitor ZM323881, which selectively inhibited VEGF stimulated VEGFR2 phosphorylation. Immunoblot results suggested ZM323881 only partly weakened the phosphorylation of VEGFR2 after treatment with FN-EDA (Fig. 3K), indicating that in addition to VEGF, there was another potential pathway through which FN-EDA could stimulate VEGFR2 phosphorylation. In conclusion, the above results provide evidence that FN-EDA promotes the phosphorylation of VEGFR2.

### FN-EDA promoted VEGFR2 phosphorylation by activating integrin receptors

**Fig. 4:**
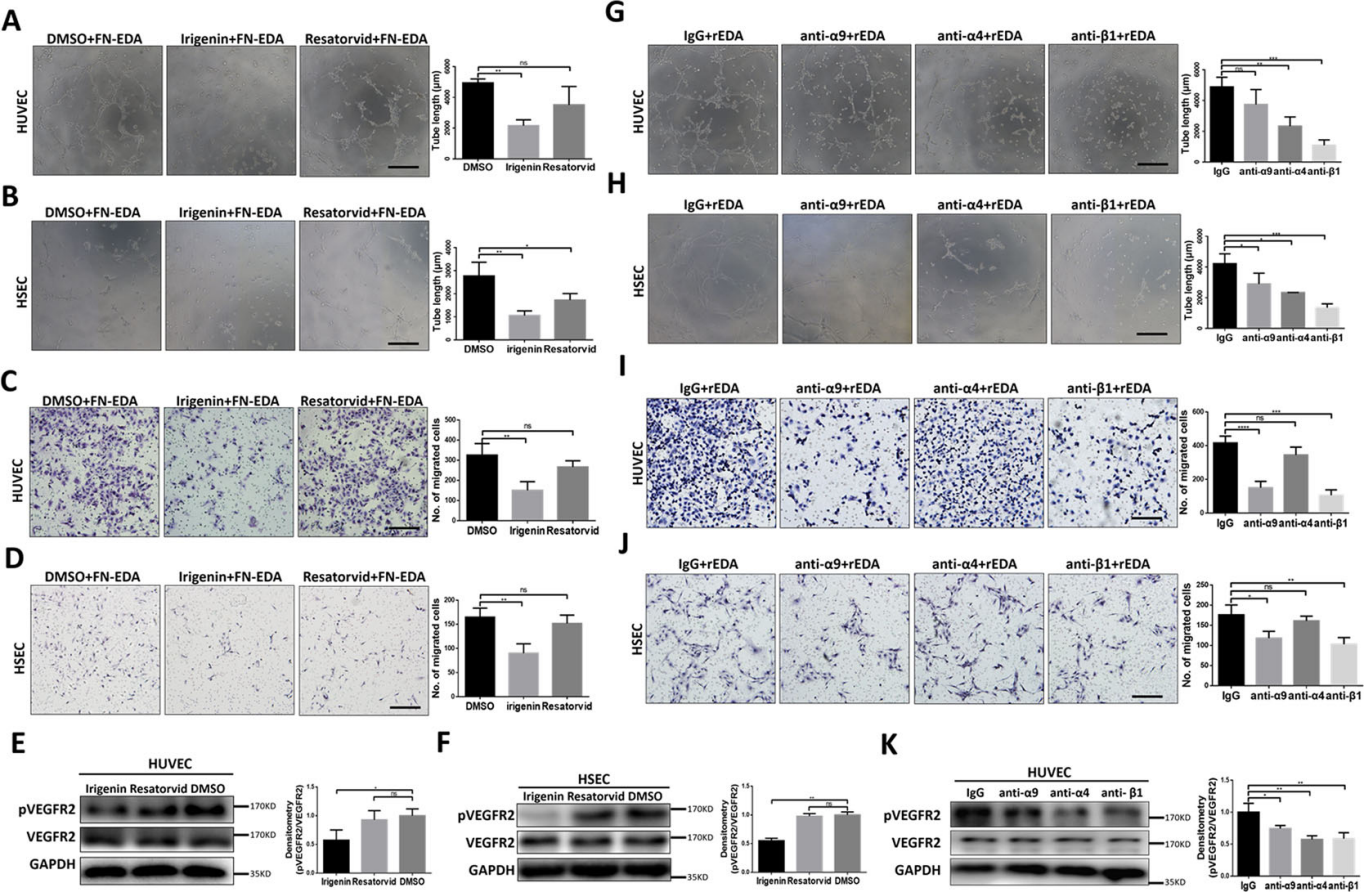
FN-EDA promoted angiogenesis through Integrins. **A**, **B** Tube formation assay of HUVECs and HSECs treated with Irigenin or Resatorvid (5 μM) along with FN-EDA (40 μg/ml) for 6 h (*n* = 3). (Data are shown as mean ± SEM; HUVEC: Irigenin versus Ctrl: **P < 0.001; Resatorvid versus Ctrl: ns. HSEC, Irigenin versus Ctrl: **P < 0.001; Resatorvid versus Ctrl: *P < 0.01; scale bar = 100 μm.) **C**, **D** Migration assay of HUVECs and HSECs treated with Irigenin or Resatorvid (5 μM) along with FN-EDA (40 μg/ml) for 24 h (*n* = 3). (Data are shown as mean ± SEM; HUVEC, Irigenin versus Ctrl: **P < 0.001; Resatorvid versus Ctrl: ns. HSEC, Irigenin versus Ctrl: **P < 0.001; Resatorvid versus Ctrl: ns; scale bar = 100 μm.) **E**, **F** Immunoblot result of HUVECs and HSECs treated with Irigenin or Resatorvid (5 μM) along with FN-EDA (40 μg/ml) for 6 h (*n* = 3). (Data are shown as mean ± SEM; HUVEC: Irigenin versus Ctrl: *P < 0.01; Restorvid versus Ctrl: ns; HSEC: Irigenin versus Ctrl: **P < 0.001; Restorvid versus Ctrl: ns.) **G**, **H** Tube formation assay of HUVECs and HSECs pretreated with varies of integrin neutralizing antibodies anti-α9, anti-α4, anti-β1 or IgG (40 μg/ml) 20 minutes before rEDA (40 μg/ml) stimulation for 6 h (*n* = 3). (Data are shown as mean ± SEM; HUVEC: anti-α9 versus Ctrl: ns; anti-α4 versus Ctrl: **P < 0.001; anti-β1 versus Ctrl: ***P < 0.0001. HSEC, anti-α9 versus Ctrl: *P < 0.01; anti-α4 versus Ctrl: *P < 0.01; anti-β1 versus Ctrl: ***P < 0.0001; scale bar = 100 μm.) **I**, **J** Migration assay of HUVECs and HSECs treated with varies of integrin neutralizing antibodies anti-α9, anti-α4, anti-β1 or mouse IgG (40 μg/ml) 20 minutes before rEDA (40 μg/ml) stimulation for 24 h (*n* = 3). (Data are shown as mean ± SEM; HUVEC, anti-α9 versus Ctrl: ***P < 0.0001; anti-α4 versus Ctrl: ns; anti-β1 versus Ctrl: ***P < 0.0001. HSEC, anti-α9 versus Ctrl: **P < 0.001; anti-α4 versus Ctrl: ns; anti-β1 versus Ctrl: *P < 0.01; scale bar = 100 μm.) **K** Immunoblot result of HUVECs pretreated with varies of integrin neutralizing antibodies anti-α9, anti-α4, anti-β1 or IgG (40 μg/ml) 20 min before rEDA (40 μg/ml) stimulation for 6 h (*n* = 3). (Data are shown as mean ± SEM; HUVEC, anti-α9 versus Ctrl: *P < 0.01; anti-α4 versus Ctrl: **P < 0.001; anti-β1 versus Ctrl: **P < 0.001).

FN-EDA can bind integrins and Toll-like receptor 4 (TLR4)^19,36^. To further explore the mechanism by which FN-EDA promotes the phosphorylation of VEGFR2, we used the small molecular inhibitor irigenin (specifically blocking the EDA segment and integrin conjunction^37^) or resatorvid (a specific TLR4 inhibitor^19^) to specifically inhibit the interaction of FN-EDA and these potential receptors. Irigenin showed an inhibitory effect in both migration and tube formation assays on HUVECs and HSECs after stimulation by FN-EDA while resatorvid showed only a limited inhibitory effect (Fig. 4A–D). Meanwhile, phosphorylation of VEGFR2 was significantly weakened by irigenin but not resatorvid which was consistent with the above results (Fig. 4E, F).

Therefore, we focused on integrin and used a series of integrin neutralizing antibodies, including anti-α4, anti-α9, and anti-β1 to explore which integrin subunit plays the core role. We used rEDA to stimulate endothelial cells that were pretreated with the above integrin neutralizing antibodies. In HUVECs and HSECs, after blocking integrin β1, both migration and tube formation were dramatically inhibited while after blocking integrin α4 only tube formation ability was significantly attenuated, and after blocking integrin α9 only migration ability was significantly decreased (Fig. 4G–J). Furthermore, we detected phosphorylation of VEGFR2 after blocking the above integrins before treated with rEDA. We observed that after blocking integrin α4 and β1, phosphorylation of VEGFR2 was significantly decreased (Fig. 4K). These results indicated that integrin receptors, especially integrin β1, were crucial for FN-EDA-promoted angiogenesis and VEGFR2 phosphorylation.

### CD63 mediated integrin and VEGFR2 coaggregation is crucial for FN-EDA induced angiogenesis

**Fig. 5:**
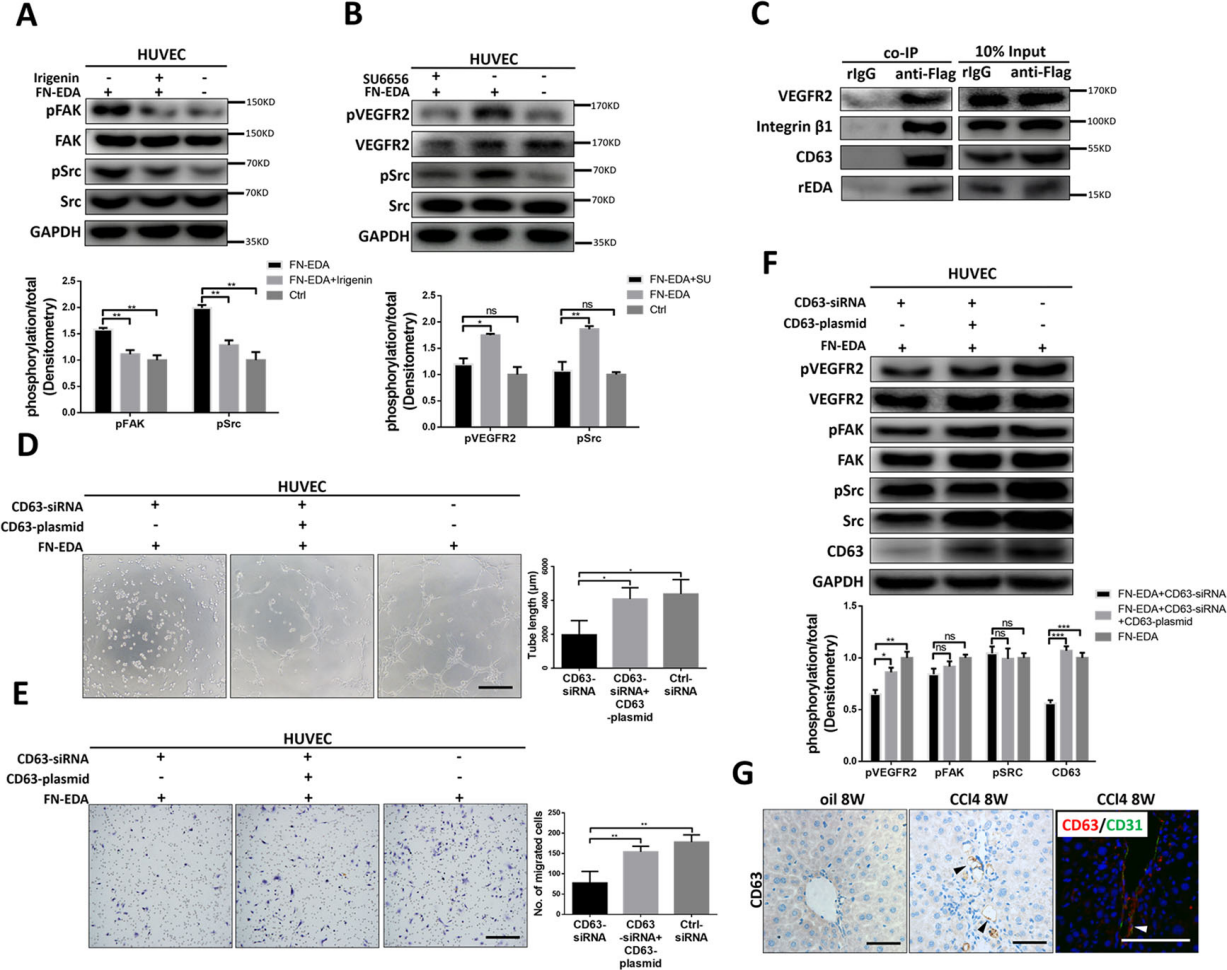
CD63 participated in FN-EDA induced angiogenesis. **A** Immunoblot result of HUVECs treated with FN-EDA and/or irigenin for 6 h. (Data are shown as mean ± SEM; pFAK: FN-EDA versus Irigenin+FN-EDA: **P < 0.001, FN-EDA versus Ctrl: **P < 0.001; pSrc: FN-EDA versus Irigenin+FN-EDA: **P < 0.001, FN-EDA versus Ctrl: **P < 0.001.) **B** Immunoblot result of HUVECs treated with FN-EDA and/or Src inhibitor SU6656 for 6 h. (Data are shown as mean ± SEM; pVEGFR2: FN-EDA + SU6656 versus FN-EDA: *P < 0.01; pSrc: FN-EDA versus Ctrl: ns; pSrc: FN-EDA + SU6656 versus FN-EDA: **P < 0.001; pSrc: FN-EDA versus Ctrl: ns.) **C** Immunoblot result of HUVECs lysis immunoprecipitated by anti-flag antibody. HUVEC were treated with rEDA (40 μg/ml) for 6 h before harvest. **D** Tube formation assay of HUVECs transfected with CD63-siRNA alone or both CD63-siRNA and CD63-plasmid before treated with FN-EDA (40 μg/ml) for 6 h (*n* = 3). (Data are shown as mean ± SEM; CD63-siRNA versus CD63-siRNA+ CD63-plasmid: *P < 0.01 CD63-siRNA versus Ctrl-siRNA: *P < 0.01; scale bar = 100 μm.) **E** Migration assay of HUVECs transfected with CD63-siRNA alone or both CD63-siRNA and CD63-plasmid before treated with FN-EDA (40 μg/ml) for 24 h (*n* = 3). (Data are shown as mean ± SEM; CD63-siRNA versus CD63-siRNA+ CD63-plasmid: **P < 0.001 CD63-siRNA versus Ctrl-siRNA: **P < 0.001. Scale bar = 100 μm.) **F** Immunoblot result of HUVECs transfected with CD63-siRNA alone or both CD63-siRNA and CD63-plasmid before treated with FN-EDA (40 μg/ml) for 6 h. (Data are shown as mean ± SEM; pVEGFR2: CD63-siRNA versus CD63-siRNA+CD63-plasmid: *P < 0.01; CD63-siRNA versus Ctrl: **P < 0.001; pFAK: CD63-siRNA versus CD63-siRNA+CD63-plasmid: ns; CD63-siRNA versus Ctrl: ns; pSrc: CD63-siRNA versus CD63-siRNA+CD63-plasmid: ns; CD63-siRNA versus Ctrl: ns; CD63: CD63-siRNA versus CD63-siRNA+CD63-plasmid: ***p < 0.0001; CD63-siRNA versus Ctrl: ***p < 0.0001.) **G** Immunohistochemical staining of CD63 in mice fibrosis hepatic tissues. Immunofluorescence shows CD63 (red) and CD31 (green) were co-stained.

Integrins are important mediators in angiogenesis and are often coactivated by receptor tyrosine kinases (RTKs), including VEGFRs^38,39^. Therefore, we detected the integrin downstream pathway and found that phosphorylation of Src and FAK was significantly increased after FN-EDA stimulation and was attenuated after inhibition by irigenin (Fig. 5A). Several studies have demonstrated transactivation of RTKs in a ligand independent manner through integrin and its downstream kinases especially Src^40–42^. Thus, we pretreated HUVECs with SU6656 (a specific Src inhibitor) before FN-EDA stimulation, and it was observed that the phosphorylation of VEGFR2 enhanced by FN-EDA was decreased (Fig. 5B).

Src is recruited to activated integrins^39^, if recruited kinases straightforward activate VEGFR2, the neighborship between integrins and VEGFR2 must be important. Tetraspanins are a family of proteins that form tetraspanin-enriched microdomains within the plasma membrane and simultaneously bind receptors, including RTKs and integrins^43^. CD63 is among the most highly expressed tetraspanins in endothelial cells^44^. Therefore, we performed co-immunoprecipitation to confirm the combination of FN-EDA with β1-CD63-VEGFR2 complex. Integrin, CD63 and VEGFR2 were co-immunoprecipitated with rEDA (Fig. 5C). Next, we knocked down CD63 expression in HUVECs and found that tube formation and migration stimulated by FN-EDA were significantly decreased, and angiogenesis abilities were recovered when CD63 was reoverexpressed (Fig. 5D, E). Similar changes were also observed in the phosphorylation of VEGFR2 while the phosphorylation of FAK and Src were barely impaired (Fig. 5F). In addition, CD63 was highly expressed in the neovessel areas and colocalized with CD31 in mouse experimental liver fibrosis models (Fig. 5G). The above results indicate that CD63, the bridge linking integrin β1 with VEGFR2 to maintain their spatial contiguity, plays an indelible role.

### Blocking of EDA/integrin combination suppresses hepatic angiogenesis and fibrosis in vivo

**Fig. 6:**
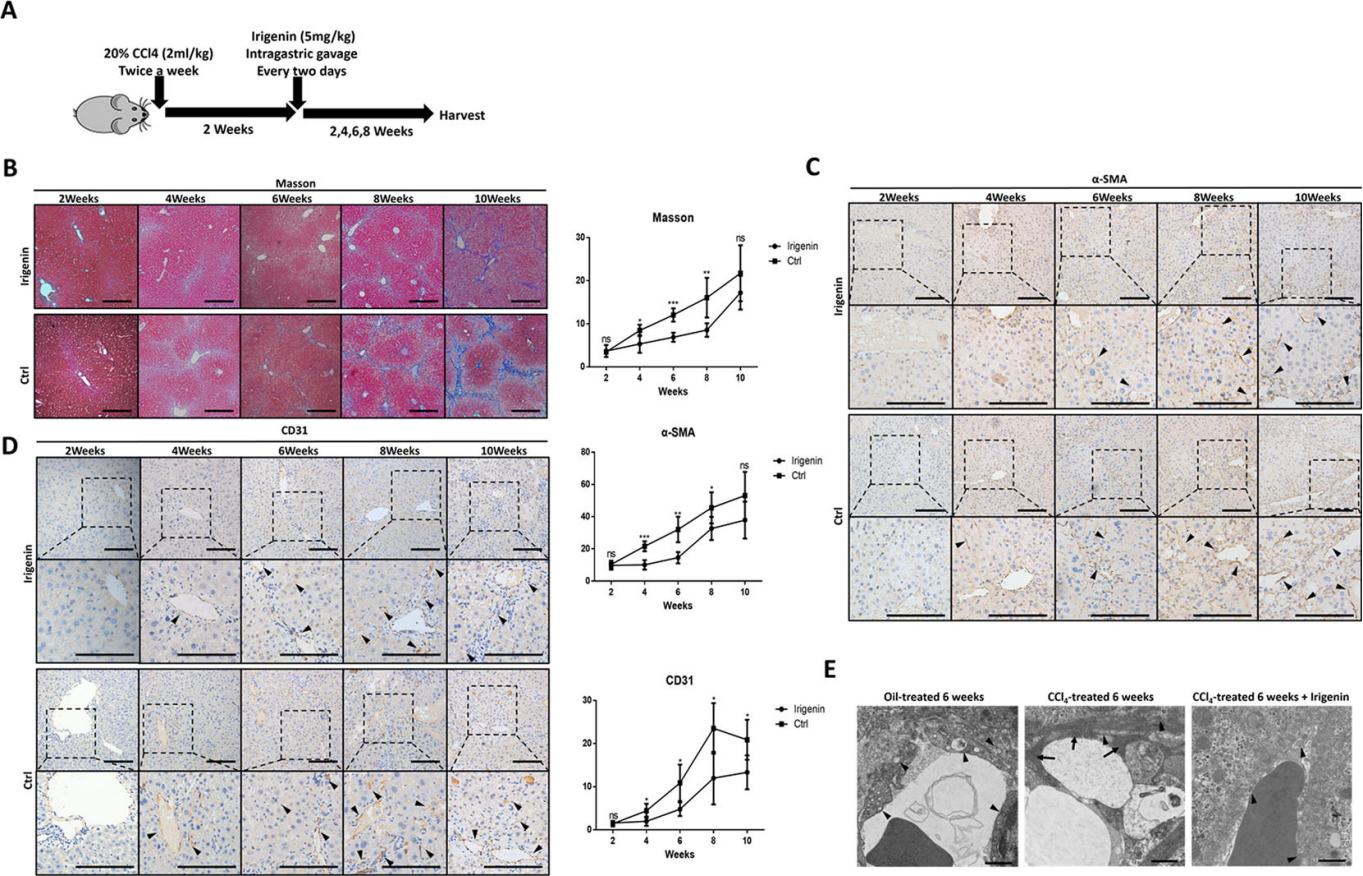
Irigenin relived mouse CCl4-induced hepatic fibrosis. **A** Schematic representation of hepatic fibrosis induction in C57BL/6 mice and treated with irigenin. **B** Masson staining in mouse CCl_4_-induced fibrotic livers treated with irigenin (*n* = 3). (Analysis relative area of Masson staining. Data are shown as mean ± SEM; week 2: ns; week 4: *P = 0.0207; week 6: ***P = 0.0003; week 8: **P = 0.0087; week 10: ns; scale bar = 200 μm.) **C** Immunohistochemical analysis of α-SMA expression in mouse CCl_4_-induced fibrotic livers treated with irigenin (*n* = 3). (Data are shown as mean ± SEM. Week 2: ns; week 4: ***P = 0.0003; week 6: **P = 0.0019; week 8: *P = 0.0436; week 10: ns; scale bar = 200 μm.) **D** Immunohistochemical analysis of CD31 expression in mouse CCl_4_-induced fibrotic livers treated with irigenin. Irigenin reduced CD31 expression in liver during hepatic fibrosis (*n* = 3). (Data are shown as mean ± SEM; week 2: ns week 4: *P = 0.0235; week 6: *P = 0.0169; week 8: *P = 0.0160; week 10: ns; scale bar = 200 μm.) **E** Transmission electron microscope (TEM) showing the hepatic sinusoids in the liver tissues. (Short arrows: LSEC fenestrae; long arrows: basement membrane; scale bar = 10 μm).

Irigenin is an active ingredient of the herbal medicine Rhizoma Belamcanda, which is officially listed in the Chinese pharmacopoeia and is widely used^45^. Considering its specificity in targeting the FN-EDA C-Cʹ loop^37^ and its known safety as a bioactive constituent of an officially-approved drug, irigenin could be a potential anti-angiogenesis drug for hepatic fibrosis. Therefore, we treated mice with irigenin via intragastric gavage every 2 days 2 weeks after the first treatment with CCl_4_ and collected liver tissue every two weeks thereafter (Fig. 6A). Immunohistochemical analysis showed that the irigenin-treated group had less CD31 and α-SMA expression and lower Masson stain scores than the control group in the early stage of fibrosis after treatment with irigenin. However, this protective effect was attenuated in the tenth weeks, and the differences in Masson stain and α-SMA expression were reduced (Fig. 6B–D). We also examined the fenestration of HSECs in six weeks CCl_4_-treated mouse hepatic tissues using transmission electron microscopy. In CCl_4_-treated mice, HSECs were capillarized with a thick basement membrane and shrunken fenestration, while the irigenin-treated group had little basement membrane and more fenestrations (Fig. 6E). The results above indicated that irigenin may be a potential targeted candidate drug to inhibit angiogenesis by blocking the binding of FN-EDA and integrin in hepatic fibrosis.

## Discussion

**Fig. 7:**
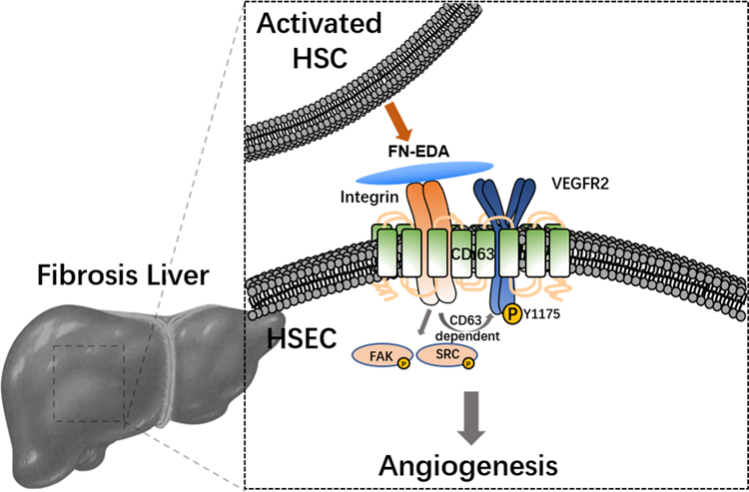
Schematic figure illustrating the mechanism of FN-EDA promoting angiogenesis in hepatic fibrosis. In hepatic fibrosis, FN-EDA is over secreted by activated HSCs to HSECs and stimulates activation of HSECs via integrin-CD63-VEGFR2 receptor complex which leading to pathological angiogenesis.

In this study, we focused on FN-EDA, a special splicing variant of fibronectin, and provided evidence to clarify its role in pathological angiogenesis and the cross-talk between HSCs and HSECs in hepatic fibrosis. We first verified the positive correlation between FN-EDA and pathological angiogenesis, and then demonstrated that FN-EDA promote angiogenesis in vitro and vivo. We observed that FN-EDA itself could promote the phosphorylation of VEGFR2, and further demonstrated the promotion effect occurred through integrin receptors and was CD63 dependent (Fig. 7). Moreover, we preliminarily verified the irigenin, which specifically blocks the conjunction of FN-EDA and integrin, as a potential anti-hepatic fibrosis therapy.

Hepatic fibrosis is characterized by excessive ECM deposition and increased intrahepatic angiogenesis which is induced by activation of HSCs and HSECs. However, the intricate interplays have not been fully understood yet. HSCs and HSECs maintain co-activation during hepatic fibrosis that not only do capillarized HSECs secret fibroblast growth factor (FGF) and transforming growth factor-β1 (TGF-β1) to facilitate the activation and ECM deposition of HSCs, but also activated HSCs paracrine pro-angiogenic factors such as VEGF, platelet-derived growth factor (PDGF) and angiopoietins to promote angiogenesis of HESCs at the same time^46–48^. Over-deposited ECM help those pro-angiogenic factors to combine with their receptors and provide scaffold for pathological angiogenesis. Previous studies indicated that ECM-anchored VEGF prolonged activation of VEGFR^49^, and ECM components provide a binding scaffold for endothelial cell anchorage and migration during angiogenesis^50^. Moreover, further studies revealed the mechanobiological mechanism of ECM in hepatic fibrosis that mechanical strain generated during ECM remodeling has been shown to mediate intrahepatic angiogenesis^51,52^.

In spite of collagen is the quantitatively dominant matrix component in many fibrosis diseases, fibronectin is an early and important component which is upregulated in many fibrotic diseases and even considered as a fibrosis marker, there were limited investigation of its specific isoform and function. FN-EDA is reportedly upregulated and participated in fibrosis process, but most of the studies focus its role on activating fibroblasts^19,20,33^. A delicate designed research indicated that FN-EDA could promote only HSC motility not activation or differentiation while CCl_4_-induced EDA KO male mice did have less fibrosis and α-SMA expression^27^. In fact, the aforementioned studies focused on the effect of exogenous FN-EDA on HSCs; however, our data, including some unpublished data, suggested that HSCs were the dominant source of FN-EDA during hepatic fibrosis and that interfering with FN-EDA expression in vitro decreased the expression of α-SMA and VEGFA but not collagen, indicating that HSC derived FN-EDA, rather than exogenous FN-EDA, is important to maintain HSC activation. A recent study reported that specific deletion of FN-EDA in smooth muscle cells, but not in endothelial cells, reduced smooth muscle cell phenotypic switching highlighting the importance of fibroblast endogenous derived FN-EDA^33^. Traditionally, FN-EDA is known as a component of extra matrix that assembles into insoluble fibrils^53^. In current study, we confirmed that FN-EDA functions as paracrine factors that promotes the phosphorylation of VEGFR2. It was an interesting finding that maybe there is a serum level change of circulating FN-EDA which could indicated hepatic fibrosis or some vascular related complications and put us a clue of local inflammation and systemic response as FN-EDA was demonstrated as a proinflammatory factor^19^. To the best of our knowledge, no previous studies have reported the promotion of pathological angiogenesis by FN-EDA in fibrotic diseases.

As there is no evidence of FN-EDA directly binding with VEGFR2, we targeted the reported receptors of FN-EDA to screen. The EDA segment has been recognized as a ligand for integrins including α4, α9 and β1^36,54^ and an established agonist for TLR4 that considered as a damage-associated molecular pattern molecule promoting fibroinflammatory^19^. Although both types of receptors are reportedly involved in angiogenesis, integrins are more likely to be related with VEGFR2, which mediates endothelial cell adhesion and migration, as well as signaling coreceptors of the receptor tyrosine kinases^38,39^. FN is a high-molecular-weight protein that has many regions other than the EDA segment that can bind with integrins^15^. To eliminate this interference, we used rEDA as previous reported^19^ to stimulate endothelial cells along with integrin neutralizing antibodies. Preliminary screening results supported our hypothesis. Further study, we systematically measured various integrin receptors and screened out the core integrin subunit β1. We also determined two auxiliary integrin subunits α4, α9, the former of which was closely related to tube formation, while the latter were more correlated with migration in our results.

The compact spatial structure of membrane proteins is critical important for signal transduction. the transmembrane-4 glycoprotein superfamily are well known for interacting among themselves and with other transmembrane proteins to form membrane microdomains^55^. CD63 is the most highly expressed tetraspanin in endothelial cells and was reported for integrin β1-VEGFR2 complex formation^56^. Therefore, to confirm the necessity of the neighborship of β1 and VEGFR2, we knocked down CD63 expression and observed attenuation of VEGFR2 phosphorylation, which was recovered after reoverexpressing CD63. These results indicated the importance of spatial relationships for transmembrane proteins cooperating with each other.

Although the importance of intrahepatic angiogenesis in hepatic fibrosis has been reported and several anti-angiogenic treatments have even been reported on hepatic fibrosis^7,9^, none has been approved for clinical use due to their low specificity and potential adverse effects. FN-EDA is expected to be a potential therapeutic target due to its strict expression in healthy adults. Some studies even identified FN-EDA as a drug delivery target and obtained good feedback which indirectly reflects the high specificity of FN-EDA to the pathological context^57^. Irigenin is one of the most abundant bioactive ingredients of isoflavones extracted from Rhizoma Belamcanda and specifically targets integrin α9β1 and α4β1 binding sites on FN-EDA in its C-Cʹ loop, inhibiting FN-EDA-induced metastasis in lung cancer^37^. Our results suggested irigenin could relieve intrahepatic angiogenesis and fibrosis in the early stage while these curative effects were weakened in the later stage, which was consistent with early studies that anti-angiogenesis therapy was more effective in the early stage of hepatic fibrosis^51^. Collectively, these data highlight the significance of FN-EDA as a potential anti-angiogenesis therapeutic target for the treatment of liver fibrosis.

## Materials and methods

### Human specimens

Liver tissue specimens with histologically diagnosed fibrosis were obtained from surgical surplus or biopsies. Normal liver tissues from the surgical surplus of liver trauma or hepatic hemangioma specimens were collected as the control. The demographic and clinical characteristics corresponding to the two groups are shown in Table 1. The authors obtained informed consent from each participant to conduct this study. All procedures were approved by the Ethics Committee of Shandong Provincial Hospital, Cheeloo College of Medicine, Shandong University (Jinan, China).

**Table 1:** Demographic and clinical characteristics of hepatic fibrosis patients and healthy individuals.

	Hepatic fibrosis patients	Healthy individuals
#	30	15
age	55.57 ± 3.40	49.40 ± 3.58
BP (mmHg)	82.00 ± 3.13 ~ 123.27 ± 5.01	78.87 ± 3.36 ~ 122.00 ± 4.62
BMI	25.14 ± 1.21	22.60 ± 0.72
PLT (109/L)	147.97 ± 29.08	233.87 ± 20.08
ALB(g/L)	39.09 ± 3.28	43.51 ± 1.71
PT (s)	14.81 ± 1.15	13.29 ± 0.27

### Mouse experiments

Six-week-old male wild-type C57BL/6 mice were purchased from the Shandong University Laboratory Animal Centre. For hepatic fibrosis model, mice were administered CCl_4_ (2 ml/kg, CCl4: olive oil = 1:4) or olive oil twice a week by intraperitoneal injection. AAV9-FN-EDA-shRNA or AAV9-Ctrl (Genechem, Shanghai, China) were injected into mice through tail vein every four weeks for eight weeks. Irigenin (5 mg/kg, Selleck, Shanghai, China) or water was administered through gavage after two weeks CCl_4_ administration. The animal study protocol was approved by the Ethics Committee of Shandong Provincial Hospital.

### Cell culture

LX-2 cells (HSC cell line) (Procell, Wuhan, CN) were cultured in DMEM with 10% FBS and 1% penicillin/streptomycin. Human umbilical vein endothelial cells (HUVECs) (ATCC, Rockville, MD, USA) were cultured in complete 1640 medium. SK-hep1 cells (Procell, Wuhan, CN) were cultured in complete MEM. Primary HSECs (ScienCell, Carlsbad, CA, USA) were cultured in complete ECM with 10% endothelial cell growth supplement (ScienCell, Carlsbad, CA, USA). For in vitro knockdown, FN-EDA-siRNAs, CD63-siRNA and control siRNA (Biosune, Jinan, CN) were transfected into HUVEC or LX-2 at 30 pmol/ml (12 well plate). For in vitro reoverexpression of CD63, the ORF of CD63 in pEnter and vector plasmid control (Biosune, Jinan, CN) were transfected at 1.5 μg/ml (12 well plate) one day after CD63-siRNA transfection each group were harvested 84 hours in total. Transient transfection was performed using Lipofectamine 3000 (Life Technologies; Gaithersburg, MD) according to the manufacturer’s protocol. The target interfering sequences are listed in Table 2.

**Table 2:** Target sequences for RNAi.

	Target sequence
FN-EDA-siRNA1	GGGACUCCUACCUUAGGUA
FN-EDA-siRNA2	CCGUAAGUGACUACACCUA
CD63-siRNA	GCUGCCUCGUGAAGAGUAU

### RNA isolation and quantitative real-time PCR (qRT-PCR)

Total RNA was extracted from human samples with TRIzol reagent, and cDNA was generated with a reverse transcription kit (Takara, Japan). cDNA was amplified by qRT-PCR on Roche 480 Real Time PCR System instrument using SYBR Green PCR kit (Takara, Japan). mRNAs were normalized to GAPDH. The primers for qRT-PCR are listed in Table 3.

**Table 3:** Primers for quantitative real-time PCR.

	Forward	Reverse
EDA	GTAACCAACATTGAT	GGCTCGAGTAGGTCAC
EDB	AGTTAGTTGCGGCAGGAGAAG	CCGCCATTAATGAGAGTGAT
CD31	CCGCATATCCAAGGTCAGCA	CACCTTGGTCCAGATGTGTGAA
GAPDH	GCACCGTCAAGGCTGAGAAC	TGGTGAAGACGCCAGTGGA

### Immunohistochemistry

Immunohistochemistry was performed as previously described^8^. Briefly, liver tissues were fixed with 4% paraformaldehyde and embedded in 5-μm-thick paraffin sections. After deparaffinization, hydration, citrate incubation, and 3% H_2_O_2_ blocking, slides were confined with 5% goat serum and incubated with primary antibodies. Following incubation with horseradish peroxidase second antibodies then reacted with a diaminobenzidine solution and counterstained with hematoxylin. The staining score was quantified by the mean integral optical density (IOD) using Image-Pro Plus 6 software (Baltimore, USA).

### Immunofluorescence

Briefly, Liver tissues were fixed with 4% paraformaldehyde and cut into 5-μm-thick sections. After being incubated with 0.01 M citrate, sections were blocked with 5% goat serum and incubated with appropriate primary antibodies. After washing by PBS, the sections were incubated with secondary antibodies conjugated with CoraLite594 or FITC, and were then counterstained with DAPI. Fluorescence score was quantified by the mean integral optical density using Image-Pro Plus 6 software.

### Immunoblot

Mouse hepatic samples were harvested in and lysed with RIPA lysis buffer supplemented with a protease and phosphatase inhibitor cocktail. After determining the protein concentration by using a BCA Protein Assay Kit (Solarbio, Beijing, CN), equal sample quantities were electrophoresed on SDS–PAGE gels and transferred onto PVDF membranes. The membrane was blocked for 1 h with 5% BSA and incubated with primary antibodies (Table 4), followed by incubation with horseradish peroxidase conjugated secondary antibodies for 1 h at room temperature. GAPDH was used as an internal control. All the antibody bands were normalized to their expression.

**Table 4:** Antibodies.

Antibody	Identifier	Application
FN-EDA	sc-59826, Santa Cruz	WB, IHC, Blocking, IF
FN-EDA	F6140, Sigma	Blocking
CD31	sc-376764, Santa Cruz	WB, IHC, IF
α-SMA	ab5694, Abcam	WB, IHC, IF
CD63	SC5275, Santa Cruz	WB, IHC
CD63	ab134045, Abcam	WB
Albumin	16475-1-AP, Proteintech	IF
pVEGFR2	ab194806, Abcam	WB
pVEGFR2	#2478, CST	WB
VEGFR2	#9698, CST	WB
VEGFR2	26415-1-AP, Proteintech	WB
PI3K p85	ab191606, Abcam	WB
pPI3K p85/p55	ab226842, Abcam	WB
AKT	#4685, CST	WB
pAKT	#4060, CST	WB
PLCγ1	#5640, CST	WB
pPLCγ1	#8713, CST	WB
ERK	#4695, CST	WB
pERK	#4370, CST	WB
Src	#2109, CST	WB
pSrc	#6943, CST	WB
FAK	#3285, CST	WB
pFAK	#3281, CST	WB
integrin β1	26918-1-AP, Proteintech	WB
GAPDH	60004-1-Ig, Proteintech	WB
integrin α9	ab27947, abcam	Blocking
integrin α4	ab25247, Abcam	Blocking
integrin β1	ab24693, Abcam	Blocking
flag	#14793, CST	IP

### Tube formation assay

The tube formation assay was performed as previously described^8^. Briefly, endothelial cells were seeded at a density of 1–2 × 10^4^ cells/well for 6 h in plates precoated with Matrigel at 37 °C and 5% CO_2_. For coculturing experiment, LX-2 cells were seeded into the upper chambers of a transwell plate (Corning, Costar 3422) for 24 h and then transferred to other plates with endothelial cells. Experiments were performed in the presence or absence of various fibronectins, including pFN (40 μg/ml, ECM001, Sigma-Aldrich; St. Louis, MO, USA), FN-EDA (40 μg/ml, F2518, Sigma-Aldrich; St. Louis, MO, USA), rEDA (40 μg/ml Flag-tagged, Daian, Wuhan, China) or inhibitors including irigenin (5 μM Selleck, Shanghai, China) and, resatorvid (5 μM, MCE, USA), or neutralizing antibodies (Table 4). Tube formation was photographed using inverted microscope and quantified by calculating the average tube length using ImageJ software (National Institutes of Health, Bethesda, MD, USA)^58^.

### Transwell migration assay

Cell migration was measured by transwell assays as previously described^8^. Briefly, for coculturing experiment, we first transferred the upper chambers of the transwell plate (Corning, Costar 3422) into a 24-well plate in which LX-2 cells were seeded first, and then endothelial cells were added into the upper chambers. A total of 10^4^ cells were added to the upper side of each insert and incubated for 24 h at 37 °C and 5% CO_2_. The number of cells that migrated to the lower surface of the chamber was evaluated. Experiments were performed in the presence or absence of various factors as described above. An average of three individual wells was quantified using Image-Pro Plus software (Media Cybernetics, Madrid, USA).

### Co-immunoprecipitation

Cells were pretreated with rEDA (Flag-tagged, Daian, Wuhan, China) in 6-well plates and lysed using 1 ml of lysis buffer for IP (Beyotime) on ice for 30 min. After centrifuging at 10,000 × *g* for 15 min at 4 °C, the supernatant was incubated with flag antibody for 1 h, followed by incubation with protein A/G PLUS-Agarose beads (sc-2003, Santa Cruz) overnight, and then beads were collected by centrifugation at 2500 × *g* for 5 min at 4 °C. After washing four times with the above cell lysis buffer, beads were boiled in 5× loading buffer for 5 min followed by immunoblotting.

### Transmission electron microscopy (TEM)

Mouse liver tissues were cut into 1mm^3^ section and immersed in precooled 2.5% glutaraldehyde immediately after being separated from enterocoelia for 4 h at 4 °C and, fixed with 1% OsO4 in 0.1 M PBS (pH 7.4) for 2 h in the dark. After dehydrating by a gradient concentration of ethanol and resin penetration and embedded, the embedding models with resin and samples were moved into a 65 °C oven to polymerize for 48 h. Resin blocks were cut into 60 nm thick blocks followed by 2% uranium acetate saturated alcohol solution avoid light staining for 8 min and 2.6% lead citrate to avoid CO_2_ staining for 8 min. Images were obtained using Hitachi HT7800 transmission electron microscope.

### Statistical analysis

All statistical analyses were performed with GraphPad Prism 7.0 (La Jolla, CA). Experiments were repeated at least three independent times. Data for each group were expressed as the means ± SEM. The differences between groups were analyzed by paired and unpaired two-tailed Student’s *t* test or by ANOVA, as deemed appropriate. The Spearman rank correlation coefficients of determination were used to analyze the degree of correlation among parameters. For all analyses, the *p*-value reported was two-tailed, and *p*-values < 0.05 were considered statistically significant.
